# Alterations in the Serum Proteome Following Electroconvulsive Therapy for a Major Depressive Episode: A Longitudinal Multicenter Study

**DOI:** 10.1016/j.bpsgos.2022.11.005

**Published:** 2022-12-12

**Authors:** Andreas Göteson, Caitlin C. Clements, Anders Juréus, Erik Joas, Jessica Holmén Larsson, Robert Karlsson, Axel Nordenskjöld, Erik Pålsson, Mikael Landén

**Affiliations:** a Department of Psychiatry and Neurochemistry, Institute of Neuroscience and Physiology, University of Gothenburg, Gothenburg, Sweden; b Department of Medical Epidemiology and Biostatistics, Karolinska Institutet, Stockholm, Sweden; c Laboratories of Cognitive Neuroscience, Boston Children’s Hospital, Boston, Massachusetts; d Faculty of Medicine and Health, Örebro Universitet, Örebro, Sweden

**Keywords:** Biomarker, Depression, ECT, Longitudinal, Major depressive episode, Proteomics

## Abstract

**Background:**

Electroconvulsive therapy (ECT) is the most effective treatment for severe depression, but the biological changes induced by ECT remain poorly understood.

**Methods:**

This study investigated alterations in blood serum proteins in 309 patients receiving ECT for a major depressive episode. We analyzed 201 proteins in samples collected at 3 time points (T): just before the first ECT treatment session (T0), within 30 minutes after the first ECT session (T1), and just before the sixth ECT session (T2).

**Results:**

Using statistical models to account for repeated sampling, we identified 152 and 70 significantly (<5% false discovery rate) altered proteins at T1 and T2, respectively. The most pronounced alterations at T1 were transiently increased levels of prolactin, myoglobin, and kallikrein-6. However, most proteins had decreased levels at T1, with the largest effects observed for pro-epidermal growth factor, proto-oncogene tyrosine-protein kinase Src, tumor necrosis factor ligand superfamily member 14, sulfotransferase 1A1, early activation antigen CD69, and CD40 ligand. The change of several acutely altered proteins correlated with electric current and pulse frequency in a dose-response–like manner. Over a 5-session course of ECT, some acutely altered levels were sustained while others increased, e.g., serine protease 8 and chitinase-3-like protein 1. None of the studied protein biomarkers were associated with clinical response to ECT.

**Conclusions:**

We report experimental data on alterations in the circulating proteome triggered by ECT in a clinical setting. The findings implicate hormonal signaling, immune response, apoptotic processes, and more. None of the findings were associated with clinical response to ECT.

Depressive disorders affect millions of people every year and incur substantial societal costs (1). Strong evidence supports the use of electroconvulsive therapy (ECT) in severe depression resistant to antidepressants, particularly when psychotic symptoms or suicidal ideation are present (2, 3, 4). ECT yielded an overall clinical response rate of 80% in a large Swedish study (5,6).

Despite the effectiveness and long history of ECT, the mechanism of action remains unknown. A wide array of hypotheses has been suggested, involving hormones, neurotrophic factors, and neurotransmitters [see reviews (7, 8, 9)]. These hypotheses are based on previous studies demonstrating that ECT results in release of pituitary hormones (10), alters levels of neurotropic factors (11), and affects neuroinflammation (12) and neurotransmitters such as serotonin, glutamate, GABA (gamma-aminobutyric acid), norepinephrine, and neuropeptides (13). If better understood, biochemical changes caused by ECT may reveal novel insights into the pathomechanisms of depression as well as point to new potential pharmacological targets.

Changes in circulating levels of blood proteins offer a window into the biological mechanisms underlying ECT. Previous studies have reported ECT-related changes in a few selected proteins, which include conflicting findings on brain-derived neurotrophic factor (9,14, 15, 16); acutely increased but long-term decreased levels of interleukin 6 (IL-6) and tumor necrosis factor α (TNF-α), suggesting a normalization of depression-related microglial activation (17); and unaltered levels of S100B and neuron-specific enolase, suggesting absence of significant neuronal damage (18, 19, 20). Yet, only 2 small studies have explored a broader set of the blood serum proteome in the context of ECT (21,22). Proteomic techniques have been used to study the pharmacological response of both antidepressants (23,24) and ketamine (25), and findings from other fields have demonstrated the potential of proteomics to both unravel novel biology [e.g., in Alzheimer’s disease (26)] and develop clinically viable biomarker panels [e.g., in ovarian cancer (27)].

Although ECT is very effective on average, some patients show suboptimal response to ECT, and biomarkers that predict the outcome of ECT could be leveraged to adapt a personalized treatment strategy. Increased volume in the dentate gyrus (28), differential trajectories in IL-8 blood plasma concentrations (29), and common genetic variants (30) have independently been suggested as predictors of clinical response to ECT. However, the predictive accuracy of these suggested biomarkers falls short in comparison with clinical predictors, and more exploratory biomarker studies are needed.

In the largest study to date, we employed proximity extension assay to investigate changes in circulating levels of 201 unique blood serum proteins triggered by ECT. We also studied proteins in relation to treatment response. By analyzing samples in a repeated measures design, we not only replicated some previous findings but also unraveled novel biological processes altered during the course of ECT.

## Methods and Materials

### Patients

Characteristics of the study cohort are presented in Table 1. Study participants (*N* = 309, age range 18–86 years) were patients with a major depressive episode (MDE) scheduled for an index ECT series (3 sessions per week with a minimum of 6 planned sessions) recruited at 7 hospitals in Sweden (Danderyd, Huddinge, Hudiksvall, Göteborg, Umeå, Uppsala, and Örebro). The study was conducted from 2013 to 2017. All participants provided oral and written informed consent. The study was approved by the Ethical Review Board in Stockholm, Sweden. Given that the study objective was to investigate within-subject effects of ECT on serum protein levels, we did not include a healthy control group or patients with an MDE treated with other modalities [cf. (31,32)].

**Table 1:** Baseline Characteristics

Characteristics	Overall, *n* = 260	NA
Total Sample, *n* (%)		
T0	260 (100.0%)	–
T1	257 (98.8%)	–
T2	256 (98.5%)	–
Total Number of Treatment Sessions, Median (Range)	8 (6–23)	–
Age, Years, Median (IQR)	45.0 (33.0, 59.0)	–
Female Sex, *n* (%)	162 (62.3%)	–
Indication, *n* (%)		
Bipolar disorder, depressive episode (F313, F314, F315)	36 (13.8%)	–
MDE (F320, F321, F322, F323, F329, F331, F332, F333, F339, F530)	204 (78.5%)	–
Missing indication and MADRS-S >19	9 (3.5%)	–
Other (F259, F318, F399, F412) and MADRS-S >19	11 (4.2%)	–
Disease Severity, Median (IQR)		
MADRS-S, pretreatment	34.0 (28.0, 40.0)	45
CGI-Severity, pretreatment	5.0 (4.0, 6.0)	6
CGI-Improvement	2.0 (2.0, 2.0)	30
Treatment Response (CGI-I ≤2), *n* (%)	176 (76.5%)	30
Medication at Baseline, *n* (%)		11
No medication	17 (6.8%)	–
Antidepressants	193 (77.5%)	–
Lithium	34 (13.7%)	–
Valproic acid	11 (4.4%)	–
Lamotrigine	30 (12.0%)	–
Typical neuroleptics	20 (8.0%)	–
Atypical neuroleptics	88 (35.3%)	–

CGI, Clinical Global Impressions; IQR, interquartile range; MADRS-S, Montgomery–Åsberg Depression Rating Scale−Self report; MDE, major depressive episode; NA, not available; T, time.

### Diagnostic Assessments

Diagnoses were made by the referring psychiatrists and entered into the Swedish National Quality Register for ECT along with current medications and demographic data (33). For evaluation of baseline depressive symptoms, patients completed the Montgomery–Åsberg Depression Rating Scale–Self report (34,35). Clinicians rated the disease severity using the Clinical Global Impressions (CGI)-Severity scale (36). Clinicians also completed the CGI-Improvement (CGI-I) scale after the completed ECT series (*n* = 234) (36). Patients were excluded if the quality register for ECT was not completed in a timely fashion (within 10 days) (*n* = 12), they received fewer than 6 ECT sessions (*n* = 9), they received ECT for an indication other than MDE (*n* = 24), or they did not provide a sample just before the first treatment session and prior to anesthesia (time [T] 0) (*n* = 4). The final analyzed cohort (*n* = 260) included only patients receiving ECT for MDE within the context of 1) major depressive disorder (ICD-10 codes F320, F321, F322, F323, F329, F331, F332, F333, F339, or F530); 2) bipolar disorder (F313, F314, or F315); 3) another mood disorder (F318, F399, F412, or F259) with a pretreatment Montgomery–Åsberg Depression Rating Scale–Self report score ≥20; or 4) patients missing specific indication but where MDE was indicated in free text or implied by a pretreatment Montgomery–Åsberg Depression Rating Scale–Self report score ≥20 (see Table 1). ECT was administered according to Swedish guidelines (37). Unilateral placement is standard, and age-based dosing is used with modifications throughout the treatment series according to clinical effects and seizure quality. ECT parameters from this study are summarized in Table S1 in Supplement 2. In brief, unilateral administration was used for most cases (*n* = 242, 93%) and the most frequent induction agents were thiopental (*n* = 163, 63%) and propofol (*n* = 95, 37%).

### Study Design

**Figure 1:**
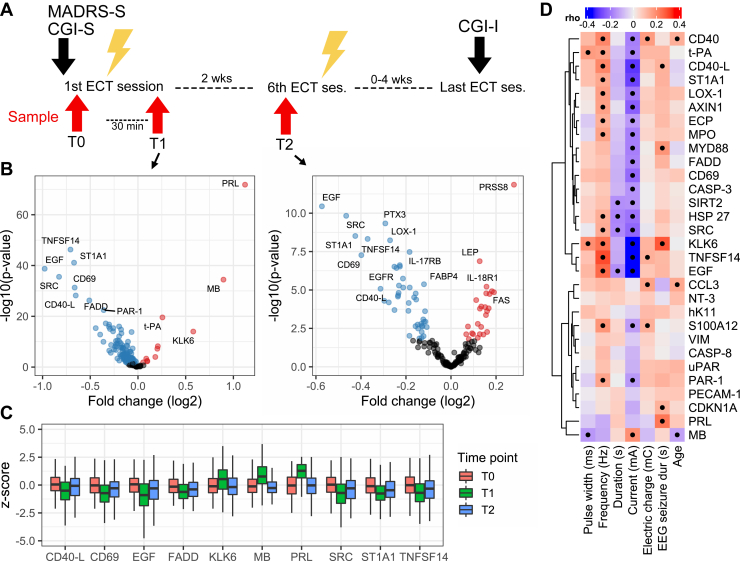
Overview of study design and protein differential abundance analysis. **(A)** Study participants were recruited for an index ECT series (2–3 weekly sessions) to treat a major depressive episode. Information on depression severity (MADRS-S and CGI-S) was collected at baseline and after the completed ECT series (CGI-I). Samples were drawn at the beginning of the first treatment session (T0), 30 minutes after the first treatment session (T1), and at the beginning of the sixth treatment session (T2). Patients continued the index ECT series between T1 and T2. **(B)** Volcano plot showing fold changes (log_2_) and *p* values from the generalized least squares models at T1 and T2, respectively. The top altered protein assays are labeled. **(C)** Boxplots showing the 10 top assays with proteins levels at each time point. **(D)** Heatmap of rank correlations between ECT parameters (including age) and the fold changes at T1 for the top 30 altered protein assays (dots indicate *p* < .05). CASP-3, caspase-3; CASP-8, caspase-8; CCL3, C-C motif chemokine 3; CDKN1A, cyclin-dependent kinase inhibitor 1; CD40-L, CD40 ligand; CD69, early activation antigen CD69; CGI-I, Clinical Global Impressions-Improvement; CGI-S, Clinical Global Impressions-Severity; ECP, eosinophil cationic protein; ECT, electroconvulsive therapy; EEG, electroencephalogram; EGF, pro-epidermal growth factor; EGFR, EGF receptor; FABP4, fatty acid binding protein 4; FADD, FAS-associated death domain protein; FAS, tumor necrosis factor receptor superfamily member 6; hK11, kallikrein-11; HSP 27, heat shock protein beta-1; IL-17RB, interleukin 17 receptor beta; IL-18R1, IL-18 receptor 1; KLK6, kallikrein-6; LEP, leptin; LOX-1, oxidized low-density lipoprotein receptor 1; MADRS-S, Montgomery–Åsberg Depression Rating Scale–Self report; MB, myoglobin; MPO, myeloperoxidase; MYD88, myeloid differentiation primary response protein MyD88; NT-3, neurotrophin-3; PAR-1, proteinase-activated receptor 1; PECAM-1, platelet endothelial cell adhesion molecule; PRL, prolactin; PRSS8, serine protease 8; PTX3, pentraxin-related protein PTX3; ses., session; SIRT2, NAD-dependent protein deacetylase sirtuin-2; SRC, proto-oncogene tyrosine-protein kinase Src; ST1A1, sulfotransferase 1A1; S100A12, protein S100-A12; t-PA, tissue-type plasminogen activator; TNFSF14, tumor necrosis factor ligand superfamily member 14; T0/T1/T2, sample time points; uPAR, urokinase plasminogen activator surface receptor; VIM, vimentin.

The study was designed to investigate acute and longitudinal effects of ECT. Blood samples were drawn at 3 different time points (Figure 1A): just before the first treatment session and prior to anesthesia (T0); within 30 minutes after the first treatment (T1); and just before the sixth treatment and prior to anesthesia (T2). Using this design, changes in protein levels from T0 to T1 reflect short-term alterations after an ECT session, whereas changes from T0 to T2 reflect longer-term changes in the blood serum proteome, occurring during the course of treatment but unrelated to the immediate effects of an ECT administration. Samples were collected in the morning with patients fasting. T2 was sampled prior to the sixth session because 6 is a frequent minimum number of sessions, and this design has been previously employed (21,31). T2 occurred at least 48 hours after the fifth ECT session. To sample the very last session, one would need to either sample each session or know the total number of sessions in advance; this number is based on an evolving clinical need and thus is unknown in clinical practice. All patients in the final cohort hence received at least 6 ECT sessions (median [range] = 8 [6–23]).

### Sample Collection

Blood samples were drawn in 10-mL serum tubes (Becton, Dickinson and Company), coagulated for 30 to 60 minutes at room temperature, and subsequently centrifuged for 15 minutes at 2000*g*. Blood serum aliquots were stored locally at the participating hospitals at −20 °C for a maximum of 30 days pending transport and storage at −70 °C at the Karolinska Institutet Biobank. Nearly all 309 included participants provided samples at all 3 time points, totaling 914 samples. Thirteen samples were missing from 11 patients (*n*_T0_ = 4, *n*_T1_ = 4, *n*_T2_ = 5).

### Multiplex Immunoassays

We analyzed blood serum levels of 201 unique proteins using 3 Olink Proteomics panels (Inflammation v.3001, CVD I v.2002, and Oncology I v.4001). These proximity extension assays combine the interaction of 2 specific antibodies with a real-time quantitative polymerase chain reaction readout. This method allows for multiplex analysis of a large number of assays with low levels of interfering crosstalk (38). Samples were analyzed in 2 waves (*n* = 329 and *n* = 585). In each wave, samples were randomized across plates (4 plates in wave 1 and 7 plates in wave 2) comprising up to 92 samples per plate in a random distribution of samples from all 3 time points.

### Preprocessing and Quality Control

Initial preprocessing and quality control were conducted by Olink Proteomics, and data were delivered in the normalized protein expression (NPX) format (39). The NPX values represent relative protein abundance on a log_2_ scale: a 1-unit increase in NPX corresponds to doubling the absolute concentration of an analyte. Additionally, 5 clear outlier samples were removed in a combined assessment based on Olink’s internal quality control steps (https://www.olink.com/resources-support/white-papers-from-olink/), principal component analysis scores, and extreme outlier values (NPX < −10 × interquartile range). Finally, the BDNF (brain-derived neurotrophic factor) assay was excluded due to technical issues.

The interpanel correlations for assays represented on multiple panels (*n* = 65) were large (median [interquartile range] *r* = 0.94 [0.91, 0.96]). We therefore discarded values from duplicate assays on the panels with the most quality control flags. Assays with > 30% of values below the limit of detection (39) at ≥ 2 time points were also excluded. Finally, the waves were combined and values were scaled with T0 values as the reference (mean_T0_ = 0, SD_T0_ = 1). The final dataset comprised 180 unique proteins passing quality control in 260 patients (*n* = 773 samples). Table S2 in Supplement 2 lists all studied proteins.

### Statistical Analyses

The statistical analyses served 2 main objectives: 1) to estimate changes in protein concentration from baseline (i.e., T0) to T1 and from T0 to T2 and 2) to identify changes in protein concentration indicative of treatment response. To estimate changes from baseline, we employed a generalized least squares (gls) model to estimate the mean value across time for each protein assay. We used an unstructured covariance pattern, i.e., allowing both variance and covariance to be estimated freely across the 3 measurement points, to account for the longitudinal nature of the data (40). The models were fitted with time, age, sex, and plate number as covariates. The percent change from baseline was estimated by 100 × (2^β^ − 1), where β is the estimate from gls models (log_2_-scale) at each time point. To investigate treatment response, we added an interaction term of treatment response and time. Treatment response was dichotomized from clinicians’ CGI-I ratings (see Results). *p* Values from the gls models were adjusted using the false discovery rate (FDR) (41) where FDR <5% was considered significant. Finally, to test whether specific ECT parameters from the first treatment session influenced the protein levels at T1, we analyzed rank correlations between individual ECT parameters and the estimated mean values at T1 from the gls models.

To explore converging pathways and functions, we annotated the top altered proteins using public databases [Gene Ontology (42), PANTHER (43), KEGG pathways, and STRING-db (44)]. Given the targeted set of proteins and the absence of a control group, we were unable to statistically test for pathway enrichment. All analyses were conducted in R (v.4.1.1) (45) using external packages: nlme (v.3.1), tidyverse (v.1.3.1), and Hmisc (v.4.5). The code is available at http://github.com/andreasgoteson.

## Results

### Protein Differential Abundance Analyses

In 260 included individuals with MDE who underwent an index ECT series, we sampled blood serum at 3 time points to compare protein levels at baseline (T0), 30 minutes after the first ECT session (T1), and just before the sixth ECT session (T2). Out of the 180 included proteins, 152 showed significant (<5% FDR) changes in protein concentration between baseline (T0) and T1, adjusted for relevant covariates (Figure 1B). The +114% increase in prolactin was the largest change observed at T1, along with highly increased levels of myoglobin and kallikrein-6. However, the majority (*n* = 116) of the significantly altered proteins showed decreases at T1, with large effect sizes seen for pro-epidermal growth factor (EGF) (−49%), proto-oncogene tyrosine-protein kinase Src (SRC), sulfotransferase 1A1 (SULT1A1), and TNF superfamily member 14 (TNFSF14).

With regard to changes from T0 to T2, 70 proteins showed significantly altered concentrations (Figure 1C). Again, most (*n* = 50) of the significantly altered proteins showed decreased circulating levels at T2, with the largest effect sizes seen for EGF (−33%), SRC, and SULT1A1. Serine protease 8 (+21%) and chitinase-3-like protein 1 (+14%) were the top proteins with increased levels at T2. Table 2 lists the 20 proteins with the largest magnitude of change from T0 to T1 and from T0 to T2.

**Table 2:** Top 20 Proteins With The Largest Fold Change at T1 and T2, Respectively.

Protein	UniProt Accession	T1				T2			
		Estimate	Standard Error	*p* Value	FDR	Estimate	Standard Error	*p* Value	FDR
Axin-1	O15169	−0.4	0.053	1.50 × 10^−13^	1.20 × 10^−12^	−0.28	0.069	6.40 × 10^−5^	3.40 × 10^−4^
CASP-3	P42574	−0.3	0.059	3.40 × 10^−7^	1.20 × 10^−6^	−0.15	0.076	.054	.11
CD40-L	P29965	−0.65	0.056	6.50 × 10^−29^	1.50 × 10^−27^	−0.31	0.07	8.30 × 10^−6^	7.10 × 10^−5^
DNER	Q8NFT8	−0.063	0.041	.12	0.13	−0.23	0.044	2.40 × 10^−7^	4.00 × 10^−6^
CD69	Q07108	−0.66	0.054	4.90 × 10^−32^	1.30 × 10^−30^	−0.4	0.073	5.40 × 10^−8^	1.10 × 10^−6^
EGFR	P00533	−0.14	0.043	.0017	0.0026	−0.24	0.047	3.70 × 10^−7^	4.70 × 10^−6^
FADD	Q13158	−0.51	0.046	5.50 × 10^−27^	1.10 × 10^−25^	−0.3	0.073	5.10 × 10^−5^	2.80 × 10^−4^
HSP 27	P04792	−0.44	0.053	4.60 × 10^−16^	5.00 × 10^−15^	−0.18	0.071	.0096	.027
hK11	Q9UBX7	−0.3	0.035	1.70 × 10^−17^	2.40 × 10^−16^	0.046	0.048	.34	.49
KLK6	Q92876	0.58	0.073	1.00 × 10^−14^	8.50 × 10^−14^	−0.12	0.053	.028	.064
LITAF	Q99732	−0.15	0.038	1.10 × 10^−4^	2.40 × 10^−4^	−0.23	0.05	5.10 × 10^−6^	5.40 × 10^−5^
MPO	P05164	−0.3	0.036	1.50 × 10^−16^	1.80 × 10^−15^	−0.25	0.049	3.00 × 10^−7^	4.20 × 10^−6^
MB	P02144	0.9	0.069	3.70 × 10^−35^	1.10 × 10^−33^	−0.14	0.057	.014	.037
SIRT2	Q8IXJ6	−0.28	0.06	2.40 × 10^−6^	7.60 × 10^−6^	−0.027	0.082	.74	.83
OSM	P13725	−0.2	0.027	7.90 × 10^−13^	5.70 × 10^−12^	−0.24	0.056	2.70 × 10^−5^	1.70 × 10^−4^
LOX-1	P78380	−0.29	0.036	9.70 × 10^−16^	9.70 × 10^−15^	−0.27	0.046	5.80 × 10^−9^	1.50 × 10^−7^
PTX3	P26022	−0.16	0.03	6.20 × 10^−8^	2.40 × 10^−7^	−0.29	0.046	4.70 × 10^−10^	2.10 × 10^−8^
PECAM-1	P16284	−0.24	0.041	7.40 × 10^−9^	3.30 × 10^−8^	−0.23	0.044	3.00 × 10^−7^	4.20 × 10^−6^
EGF	P01133	−0.98	0.07	1.90 × 10^−39^	8.30 × 10^−38^	−0.57	0.085	3.60 × 10^−11^	3.20 × 10^−9^
PRL	P01236	1.1	0.056	1.50 × 10^−72^	2.70 × 10^−70^	−0.037	0.045	.41	.56
S100A12	P80511	−0.23	0.038	6.10 × 10^−9^	2.80 × 10^−8^	−0.23	0.053	1.50 × 10^−5^	1.20 × 10^−4^
PAR-1	P25116	−0.36	0.035	3.60 × 10^−23^	6.50 × 10^−22^	−0.13	0.041	.0023	.0079
SRC	P12931	−0.83	0.062	2.80 × 10^−36^	1.00 × 10^−34^	−0.47	0.072	1.50 × 10^−10^	8.90 × 10^−9^
TGF-α	P01135	−0.11	0.035	.0022	0.0033	−0.23	0.055	2.20 × 10^−5^	1.40 × 10^−4^
PRSS8	Q16651	−0.06	0.024	.013	0.017	0.28	0.039	1.40 × 10^−12^	2.50 × 10^−10^
ST1A1	P50225	−0.67	0.046	7.70 × 10^−42^	4.60 × 10^−40^	−0.43	0.071	3.10 × 10^−9^	1.10 × 10^−7^
TNFSF14	O43557	−0.71	0.046	5.20 × 10^−47^	4.70 × 10^−45^	−0.37	0.063	4.80 × 10^−9^	1.40 × 10^−7^
CD40	P25942	−0.34	0.038	6.30 × 10^−18^	9.50 × 10^−17^	−0.12	0.047	.01	.028
VIM	P08670	−0.36	0.055	1.10 × 10^−10^	6.60 × 10^−10^	−0.25	0.067	2.00 × 10^−4^	9.10 × 10^−4^

CASP-3, caspase-3; CD40-L, CD40 ligand; DNER, Delta and Notch-like epidermal growth factor-related receptor; EGF, pro-epidermal growth factor; EGFR, EGF receptor; FADD, FAS-associated death domain protein; FDR, false discovery rate; hK11, kallikrein-11; HSP 27, heat shock protein beta-1; KLK6, kallikrein-6; LITAF, lipopolysaccharide-induced tumor necrosis factor-alpha factor; LOX-1, oxidized low-density lipoprotein receptor 1; MB, myoglobin; MPO, myeloperoxidase; OSM, Oncostatin-M; PAR-1, Proteinase-activated receptor 1; PECAM-1, Platelet endothelial cell adhesion molecule; PRL, prolactin; PTX3, pentraxin-related protein PTX3; PRSS8, serine protease 8; S100A12, protein S100-A12; SIRT2, NAD-dependent protein deacetylase sirtuin-2; SRC, proto-oncogene tyrosine-protein kinase Src; ST1A1, sulfotransferase 1A1; T, time; TGF-α; pro-transforming growth factor α; TNFSF14, TNF superfamily member 14; VIM, vimentin.

### Association With ECT Parameters

We next explored the influence of ECT parameters on the magnitude of change at T1 with the hypothesis that a higher fold change would correlate with applied current. Indeed, for most of the proteins with significantly altered levels at T1, we found a significant (*p* < .05) correlation with electric current in the expected direction (Figure 1D). Some notable exceptions include kallikrein-6, which increased at T1 but correlated negatively with electric current, and prolactin, which only correlated significantly with electroencephalogram seizure duration.

### Associations With Treatment Response

To identify proteins associated with clinical treatment response, we investigated the effect of response as well as the interaction effect of time and response on T1 and T2 protein levels. Treatment response was defined by clinicians’ rating of CGI-I, where a score of 1 or 2 (“very much improved” or “much improved,” respectively) was considered response (*n* = 180, 79%) and all other records were considered nonresponse (*n* = 51, 21%; CGI-I scores missing from 30 individuals). The largest effect sizes per term were a negative response estimate for lymphotoxin-alpha (estimate [SE] = −0.4 [0.14], *p* = .0035), a negative interaction effect with Parkinson’s disease protein 7 at T1 (estimate [SE] = −0.36 [0.16], *p* = .025), and a negative interaction effect of oncostatin-M at T2 (estimate [SE] = −0.28 [0.13], *p* = .038). However, none of the associations survived correction for multiple testing (5% FDR). Complete results, as well as estimated response curves, are presented in Table S5 in Supplement 2 and Figures S1A–E in Supplement 1.

### Functional Annotations

Finally, we annotated proteins with a significant (5% FDR) change of more than ±10% from baseline to functional databases to explore converging functions and pathways (Figures S2–S6 in Supplement 1). Most differentially abundant proteins were either intercellular signaling molecules or transmembrane signaling receptors involved in signal transduction, inflammatory response, and apoptotic processes. The biological processes associated with short-term (T0–T1) altered proteins include cell-cell signaling like immune response and TNF-mediated signaling, proteolysis, and response to mechanical stimulus and hypoxia. By contrast, proteins that changed from T0 to T2 were related to regulatory processes such as regulation of MAPK/ERK (mitogen-activated protein kinase/extracellular signal-regulated kinase) cascade and GTPase activity.

## Discussion

This study was conducted to understand how ECT affects circulating proteins in a large longitudinal sample of patients with MDE (*n* = 260) receiving ECT treatment. We sampled blood serum at the beginning of the first ECT session (T0), 30 minutes after the first session (T1), and at the beginning of the sixth session (T2). We then analyzed 201 unique proteins reflecting a broad set of biological processes. The most pronounced changes include transient increased levels of prolactin, myoglobin, and kallikrein-6, as well as decreased levels of EGF, SRC, TNFSF14, CD69, and CD40L/CD40, which were observed between T0 and T1 and partly sustained to T2. We also analyzed protein levels in relation to clinical treatment response but found no statistically significant associations. Taken together, our findings elucidate numerous biological processes altered by ECT, including pituitary hormone signaling, immune response, apoptotic processes, MAPK/ERK signal transduction, and protease activity.

To our knowledge, there are only 2 prior small studies investigating changes in the circulating blood serum proteome over an index ECT series. In line with a pilot study (*n* = 12) by Stelzhammer *et al.* (21), we report acute decreased levels of EGF, CD40-L, CD40, MMP-1, MPO, IL1-ra, S100A12, resistin, CXCL10, and CCL4, but we found inverse fold change in PGF and SCF, and did not replicate altered levels of IL-8 and CXCL9. Ryan *et al.* (22) used 2-dimensional difference in gel electrophoresis coupled with mass spectrometry and identified 36 proteins altered by ECT, of which none were included in the protein panels utilized in this study.

### Acute Effects

The most pronounced finding was a transient doubling of serum levels of prolactin, which replicates previous reports (46,47). Prolactin secreted into the bloodstream targets numerous cytokine receptors, thereby regulating various processes in reproduction, metabolism, and immune system regulation (48). In the central nervous system (CNS), prolactin interacts with the dopaminergic system (49) and has various trophic and neuroprotective effects on glial cells (50). The transient surge in prolactin is likely stress induced (48), and several other proteins involved in maintaining cellular integrity in response to stress were also altered at T1 (e.g., HSP-27, VIM, SIRT2, and CDKN1A). Myoglobin also showed a marked transient increase at T1, most likely caused by muscle contractions during seizure. Myoglobin has been suggested as a biomarker for ECT-related muscle damage (51) but was notably not correlated with electroencephalogram seizure time in our data.

Previous studies have demonstrated that ECT triggers an acute immune response (17) while our study provides greater temporal and molecular specificity. Within 30 minutes after the first ECT session (T1), we detected decreased circulating levels of proteins involved in the early stages of the immune response such as T cell activation: early activation antigen CD69 expressed by naïve T cells, costimulatory markers involved in T cell activation (i.e., CD40, CD40LG, and TNFSF14), and increased levels of IL-7 promoting T cell development in bone marrow (52). Notably, IL-6, a proinflammatory cytokine of previous interest in depression research (53), showed only a minor increase at T1 (+4%). Moreover, the hypertensive and inflammatory reaction induced by ECT might cause blood vessels to nonselectively leak plasma components into the tissue. Such leakage could explain the marginally decreased levels of most proteins at T1 compared with T0. Our findings further indicate augmented activity in the FAS signaling apoptotic pathway, which regulates the immune response (54). We detected altered circulating levels of Fas ligand and receptor as well as components of the death-inducing complex (FADD and caspase-8) and effector caspases (caspase-3), most pronounced at T1 but also partly sustained to T2.

Another group of findings involve cellular growth regulation, such as EGF signaling that is of vast importance for growth, survival, proliferation, and differentiation of many cell types (including cortical neurons). We found decreased circulating levels of EGF (−49%) together with its receptor and heterodimers (receptor tyrosine-protein kinases erbB-2 and erbB-4), again most pronounced at T1 but partly sustained to T2. EGF signaling has been associated with numerous disease processes, including schizophrenia (55) and depression (56,57). Intracellular mediators of growth signaling are also represented among the top decreased proteins at T1, such as the ubiquitous kinase SRC involved in various signaling pathways, and axin-1 involved in Wnt signaling.

### Longitudinal Effects

Over a 5-session course of ECT (i.e., from T0 to T2), some acutely triggered events were sustained but admixed with various processes involved in tissue modulation. The topmost increased protein at T2 was serine protease 8 (+21%), which cleaves and activates epithelial sodium channels and thereby regulates sodium currents that may be altered after a seizure (58). Further, proteins involved in the regulation of the acute immune response (e.g., T helper 2 cell activity and IL-13 signaling) and apoptosis of inflammatory cells were altered at T2, such as chitinase-3-like protein 1 and pentraxin-related protein PTX3.

### Biomarkers of Response to ECT

None of the tested serum proteins were significantly associated with treatment response after correction for multiple testing. We recognize 2 major limitations to this null finding. First, ECT is an efficacious treatment for MDE (79% response rate in our sample), leaving few observations for modeling nonresponse. Second, the included proteins were targeted for inflammatory, cardiovascular, and oncological disease processes and do not specifically reflect CNS processes. A few of the included proteins have distinct roles in the CNS, notably, kallikrein-6, which was increased at T1 (+49%) and is a serine protease with activity against, e.g., amyloid precursor protein (59) and alpha synuclein (60,61); sulfotransferase 1A1, which was highly decreased at T1 (−37%) and sustained to T2 and which catalyzes sulfate conjugation of many chemical compounds, including neurotransmitters (62); Parkinson’s disease protein 7, which has various neuroprotective properties (63) and showed a differential temporal trajectory in responders and nonresponders (did not pass 5% FDR); and glial-derived neurotrophic factor, a neurotrophic factor mainly for dopaminergic neurons (64), which, interestingly, was high in responders and low in nonresponders at T2 (Figure S1 in Supplement 1). As it stands, protein biomarkers for clinical response to ECT are not ready for clinical use. Future studies are encouraged.

### Limitations

The strengths of this study include the well-powered repeated measures design and the experimental method covering a broad set of the blood serum proteome. Several limitations merit consideration. First, the large sample size required a nationwide multicenter sample collection, which might have introduced batch effects by unmeasured procedural deviations across sites. Second, there are limitations inherent to the study design. The acute effects of ECT were estimated at sampling 30 minutes after the first session (T1). This time window might be too short to capture some biochemical processes [e.g., IL-6 increases only 60 minutes after a stimulus in rodents (65)]. Further, ECT requires both general anesthesia and paralysis. Thus, the T1 effects should be interpreted as reflecting the full ECT administration and not just the specific effects of applied current. Anesthesia, muscle relaxants, and seizure-related effects would not have directly influenced the effects at T2 because samples were drawn prior to anesthesia at both T0 and T2. A third consideration concerns clinical data collected at baseline, which was comprehensive in terms of psychiatric morbidity but less so for somatic conditions (e.g., body mass index was missing). Fourth, there is risk of confounding by site regarding the correlation analyses between ECT parameters and magnitude of change at T1. Finally, for ethical reasons, it is not possible to recruit a comparison cohort of patients with severe depression who have blood drawn at the same intervals without receiving any treatment. This is a limitation and we can therefore not formally prove that our results are due to the ECT. It is, however, unlikely that the pronounced changes in serum protein concentrations that we found would spontaneously occur over a short time in patients with depression.

### Conclusions

In the largest longitudinal study of ECT-related alterations in the blood serum proteome to date, we found profound acute effects triggered by 1 ECT session with findings related to signal transduction such as hormonal signaling and inflammatory response, apoptotic processes, and proteolysis. Over a 5-session course of ECT, the acute lower levels of several intercellular signaling molecules were sustained, together with altered levels of some proteins involved in regulatory processes. These findings add to the literature of peripheral effects associated with ECT. To further our understanding of the biological mechanism of ECT, future studies are encouraged to investigate CNS-specific plasma biomarkers (e.g., neurofilament light chain) and/or biomarkers from CNS tissues (e.g., cerebrospinal fluid).

## Supplementary Material

Supplementary Tables 1-5

Key Resources Table

## References

[1] Ekman M., Granström O., Omérov S., Jacob J., Landén M. (2013). The societal cost of depression: Evidence from 10,000 Swedish patients in psychiatric care. J Affect Disord.

[2] UK ECT Review Group (2003). Efficacy and safety of electroconvulsive therapy in depressive disorders: A systematic review and meta-analysis. Lancet.

[3] Pagnin D., de Queiroz V., Pini S., Cassano G.B. (2004). Efficacy of ECT in depression: A meta-analytic review. J ECT.

[4] Lisanby S.H., Devanand D.P., Prudic J., Pierson D., Nobler M.S., Fitzsimons L., Sackeim H.A. (1998). Prolactin response to electroconvulsive therapy: Effects of electrode placement and stimulus dosage. Biol Psychiatry.

[5] Nordenskjöld A., von Knorring L., Engström I. (2012). Predictors of the short-term responder rate of electroconvulsive therapy in depressive disorders--A population based study. BMC Psychiatry.

[6] Brus O., Cao Y., Gustafsson E., Hultén M., Landen M., Lundberg J. (2017). Self-assessed remission rates after electroconvulsive therapy of depressive disorders. Eur Psychiatry.

[7] Wahlund B., von Rosen D. (2003). ECT of major depressed patients in relation to biological and clinical variables: A brief overview. Neuropsychopharmacology.

[8] McCall W.V., Andrade C., Sienaert P. (2014). Searching for the mechanism(s) of ECT’s therapeutic effect. J ECT.

[9] Pinna M., Manchia M., Oppo R., Scano F., Pillai G., Loche A.P. (2018). Clinical and biological predictors of response to electroconvulsive therapy (ECT): A review. Neurosci Lett.

[10] Haskett R.F. (2014). Electroconvulsive therapy’s mechanism of action: Neuroendocrine hypotheses. J ECT.

[11] Bouckaert F., Sienaert P., Obbels J., Dols A., Vandenbulcke M., Stek M., Bolwig T. (2014). ECT: Its brain enabling effects: A review of electroconvulsive therapy-induced structural brain plasticity. J ECT.

[12] Schwieler L., Samuelsson M., Frye M.A., Bhat M., Schuppe-Koistinen I., Jungholm O. (2016). Electroconvulsive therapy suppresses the neurotoxic branch of the kynurenine pathway in treatment-resistant depressed patients. J Neuroinflammation.

[13] Baldinger P., Lotan A., Frey R., Kasper S., Lerer B., Lanzenberger R. (2014). Neurotransmitters and electroconvulsive therapy. J ECT.

[14] van Zutphen E.M., Rhebergen D., van Exel E., Oudega M.L., Bouckaert F., Sienaert P. (2019). Brain-derived neurotrophic factor as a possible predictor of electroconvulsive therapy outcome. Transl Psychiatry.

[15] Vanicek T., Kranz G.S., Vyssoki B., Fugger G., Komorowski A., Höflich A. (2019). Acute and subsequent continuation electroconvulsive therapy elevates serum BDNF levels in patients with major depression. Brain Stimul.

[16] Mindt S., Neumaier M., Hellweg R., Sartorius A., Kranaster L. (2020). Brain-derived neurotrophic factor in the cerebrospinal fluid increases during electroconvulsive therapy in patients with depression: A preliminary report. J ECT.

[17] Yrondi A., Sporer M., Péran P., Schmitt L., Arbus C., Sauvaget A. (2018). Electroconvulsive therapy, depression, the immune system and inflammation: A systematic review. Brain Stimul.

[18] Palmio J., Huuhka M., Laine S., Huhtala H., Peltola J., Leinonen E. (2010). Electroconvulsive therapy and biomarkers of neuronal injury and plasticity: Serum levels of neuron-specific enolase and S-100b protein. Psychiatry Res.

[19] Zachrisson O.C., Balldin J., Ekman R., Naesh O., Rosengren L., Ågren H., Blennow K. (2000). No evident neuronal damage after electroconvulsive therapy. Psychiatry Res.

[20] Agelink M.W., Andrich J., Postert T., Würzinger U., Zeit T., Klotz P., Przuntek H. (2001). Relation between electroconvulsive therapy, cognitive side effects, neuron specific enolase, and protein S-100. J Neurol Neurosurg Psychiatry.

[21] Stelzhammer V., Guest P.C., Rothermundt M., Sondermann C., Michael N., Schwarz E. (2013). Electroconvulsive therapy exerts mainly acute molecular changes in serum of major depressive disorder patients. Eur Neuropsychopharmacol.

[22] Ryan K.M., Glaviano A., O’Donovan S.M., Kolshus E., Dunne R., Kavanagh A. (2017). Electroconvulsive therapy modulates plasma pigment epithelium-derived factor in depression: A proteomics study. Transl Psychiatry.

[23] Filipović D., Costina V., Perić I., Stanisavljević A., Findeisen P. (2017). Chronic fluoxetine treatment directs energy metabolism towards the citric acid cycle and oxidative phosphorylation in rat hippocampal nonsynaptic mitochondria. Brain Res.

[24] Perić I., Costina V., Stanisavljević A., Findeisen P., Filipović D. (2018). Proteomic characterization of hippocampus of chronically socially isolated rats treated with fluoxetine: Depression-like behaviour and fluoxetine mechanism of action. Neuropharmacology.

[25] Weckmann K., Deery M.J., Howard J.A., Feret R., Asara J.M., Dethloff F. (2017). Ketamine’s antidepressant effect is mediated by energy metabolism and antioxidant defense system. Sci Rep.

[26] Johnson E.C.B., Carter E.K., Dammer E.B., Duong D.M., Gerasimov E.S., Liu Y. (2022). Large-scale deep multi-layer analysis of Alzheimer’s disease brain reveals strong proteomic disease-related changes not observed at the RNA level. Nat Neurosci.

[27] Enroth S., Ivansson E., Lindberg J.H., Lycke M., Bergman J., Reneland A. (2022). Data-driven analysis of a validated risk score for ovarian cancer identifies clinically distinct patterns during follow-up and treatment. Commun Med (Lond).

[28] Nuninga J.O., Mandl R.C.W., Boks M.P., Bakker S., Somers M., Heringa S.M. (2020). Volume increase in the dentate gyrus after electroconvulsive therapy in depressed patients as measured with 7T. Mol Psychiatry.

[29] Andreou B., Reid B., Lyall A.E., Cetin-Karayumak S., Kubicki A., Espinoza R. (2022). Longitudinal trajectory of response to electroconvulsive therapy associated with transient immune response & white matter alteration post-stimulation. Transl Psychiatry.

[30] Sigström R., Kowalec K., Jonsson L., Clements C.C., Karlsson R., Nordenskjöld A. (2022). Association between polygenic risk scores and outcome of ECT. Am J Psychiatry.

[31] Bouckaert F., Dols A., Emsell L., De Winter F.L., Vansteelandt K., Claes L. (2016). Relationship between hippocampal volume, serum BDNF, and depression severity following electroconvulsive therapy in late-life depression. Neuropsychopharmacology.

[32] Maffioletti E., Gennarelli M., Gainelli G., Bocchio-Chiavetto L., Bortolomasi M., Minelli A. (2019). BDNF genotype and baseline serum levels in relation to electroconvulsive therapy effectiveness in treatment-resistant depressed patients. J ECT.

[33] Nordanskog P., Hultén M., Landén M., Lundberg J., von Knorring L., Nordenskjöld A. (2015). Electroconvulsive therapy in Sweden 2013: Data from the National Quality Register for ECT. J ECT.

[34] Montgomery S.A., Asberg M. (1979). A new depression scale designed to be sensitive to change. Br J Psychiatry.

[35] Svanborg P., Åsberg M. (2001). A comparison between the Beck Depression Inventory (BDI) and the self-rating version of the Montgomery Åsberg Depression Rating Scale (MADRS). J Affect Disord.

[36] Guy W. (1976).

[37] P. Nordanskog, A. Nordenskjöld, S. Psykiatriska Föreningen (2014): ECT: Kliniska Riktlinjer [för Elektrokonvulsiv Behandling], Stockholm: Gothia Fortbildning AB.

[38] Lundberg M., Eriksson A., Tran B., Assarsson E., Fredriksson S. (2011). Homogeneous antibody-based proximity extension assays provide sensitive and specific detection of low-abundant proteins in human blood. Nucleic Acids Res.

[39] Assarsson E., Lundberg M., Holmquist G., Björkesten J., Thorsen S.B., Ekman D. (2014). Homogenous 96-plex PEA immunoassay exhibiting high sensitivity, specificity, and excellent scalability. PLoS One.

[40] Fitzmaurice G.M., Laird N.M., Ware J.H. (2011).

[41] Benjamini Y., Hochberg Y. (1995). Controlling the false discovery rate: A practical and powerful approach to multiple testing. J R Stat Soc B (Methodol).

[42] Ashburner M., Ball C.A., Blake J.A., Botstein D., Butler H., Cherry J.M. (2000). Gene ontology: Tool for the unification of biology. The Gene Ontology Consortium. Nat Genet.

[43] Thomas P.D., Campbell M.J., Kejariwal A., Mi H., Karlak B., Daverman R. (2003). Panther: A library of protein families and subfamilies indexed by function. Genome Res.

[44] Szklarczyk D., Gable A.L., Nastou K.C., Lyon D., Kirsch R., Pyysalo S. (2021). The STRING database in 2021: Customizable protein-protein networks, and functional characterization of user-uploaded gene/measurement sets. Nucleic Acids Res.

[45] (2013). R: A Language and Environment for Statistical Computing.

[46] Ohman R., Walinder J., Balldin J., Wallin L. (1976). Prolactin response to electroconvulsive therapy. Lancet.

[47] Schoretsanitis G., Cicek M., Mathur N., Sanghani S.N., Kane J.M., Petrides G. (2020). Prolactin changes during electroconvulsive therapy: A systematic review and meta-analysis. J Psychiatr Res.

[48] Bernard V., Young J., Binart N. (2019). Prolactin - A pleiotropic factor in health and disease. Nat Rev Endocrinol.

[49] Fitzgerald P., Dinan T.G. (2008). Prolactin and dopamine: What is the connection? A review article. J Psychopharmacol.

[50] Anagnostou I., Reyes-Mendoza J., Morales T. (2018). Glial cells as mediators of protective actions of prolactin (PRL) in the CNS. Gen Comp Endocrinol.

[51] Werawatganon T., Kyokong O., Charuluxananan S., Punyatavorn S. (2004). Muscular injury after succinylcholine and electroconvulsive therapy. Anesth Analg.

[52] Zúñiga-Pflücker J.C. (2004). T-cell development made simple. Nat Rev Immunol.

[53] Hodes G.E., Ménard C., Russo S.J. (2016). Integrating interleukin-6 into depression diagnosis and treatment. Neurobiol Stress.

[54] Nagata S. (1999). Fas ligand-induced apoptosis. Annu Rev Genet.

[55] Ikeda Y., Yahata N., Ito I., Nagano M., Toyota T., Yoshikawa T. (2008). Low serum levels of brain-derived neurotrophic factor and epidermal growth factor in patients with chronic schizophrenia. Schizophr Res.

[56] Memon A.A., Sundquist K., Ahmad A., Wang X., Hedelius A., Sundquist J. (2017). Role of IL-8, CRP and epidermal growth factor in depression and anxiety patients treated with mindfulness-based therapy or cognitive behavioral therapy in primary health care. Psychiatry Res.

[57] Jacobs J.M., Traeger L., Eusebio J., Simon N.M., Sequist L.V., Greer J.A. (2017). Depression, inflammation, and epidermal growth factor receptor (EGFR) status in metastatic non-small cell lung cancer: A pilot study. J Psychosom Res.

[58] Nardone R., Brigo F., Trinka E. (2016). Acute symptomatic seizures caused by electrolyte disturbances. J Clin Neurol.

[59] Magklara A., Mellati A.A., Wasney G.A., Little S.P., Sotiropoulou G., Becker G.W., Diamandis E.P. (2003). Characterization of the enzymatic activity of human kallikrein 6: Autoactivation, substrate specificity, and regulation by inhibitors. Biochem Biophys Res Commun.

[60] Iwata A., Maruyama M., Akagi T., Hashikawa T., Kanazawa I., Tsuji S., Nukina N. (2003). Alpha-synuclein degradation by serine protease neurosin: Implication for pathogenesis of synucleinopathies. Hum Mol Genet.

[61] Tatebe H., Watanabe Y., Kasai T., Mizuno T., Nakagawa M., Tanaka M., Tokuda T. (2010). Extracellular neurosin degrades α-synuclein in cultured cells. Neurosci Res.

[62] Coughtrie M.W. (2002). Sulfation through the looking glass—Recent advances in sulfotransferase research for the curious. Pharmacogenomics J.

[63] Ariga H., Takahashi-Niki K., Kato I., Maita H., Niki T., Iguchi-Ariga S.M.M. (2013). Neuroprotective function of DJ-1 in Parkinson’s disease. Oxid Med Cell Longev 2013.

[64] Lin L.F., Doherty D.H., Lile J.D., Bektesh S., Collins F. (1993). GDNF: A glial cell line-derived neurotrophic factor for midbrain dopaminergic neurons. Science.

[65] Kakizaki Y., Watanobe H., Kohsaka A., Suda T. (1999). Temporal profiles of interleukin-1beta, interleukin-6, and tumor necrosis factor-alpha in the plasma and hypothalamic paraventricular nucleus after intravenous or intraperitoneal administration of lipopolysaccharide in the rat: Estimation by push-pull perfusion. Endocr J.

