# Supplementary material for: Alterations in the Serum Proteome Following Electroconvulsive Therapy for a Major Depressive Episode: A Longitudinal Multicenter Study
Source: Biol Psychiatry Glob Open Sci. 2022 Dec 12;3(4):884–92. doi: 10.1016/j.bpsgos.2022.11.005 (PMC10593865; doi:10.1016/j.bpsgos.2022.11.005)
Supplement: Supplementary Information [file mmc1.pdf]

SUPPLEMENTARY INFORMATION

Alterations in the Serum Proteome Following Electroconvulsive Therapy for a Major Depressive Episode: A Longitudinal Multicenter Study

Göteson *et al.*

Table of contents

Page 2-7	Supplementary Figures 1a-e. Estimated response curves for all included proteins (alphanumeric order). Showing estimated mean and standard error of the mean stratified by response.
Page 8-10	Supplementary Figures 2-6. Functional annotation of the differentially abundant proteins at T1 and T2. Proteins with more than ±10% change from baseline (5% FDR) were annotated to PANTHER protein class, Gene Ontology (GO) biological process, molecular function, and cellular component, as well as KEGG pathways. Terms annotated to <5 proteins at both T1 and T2 were filtered out in all plots.

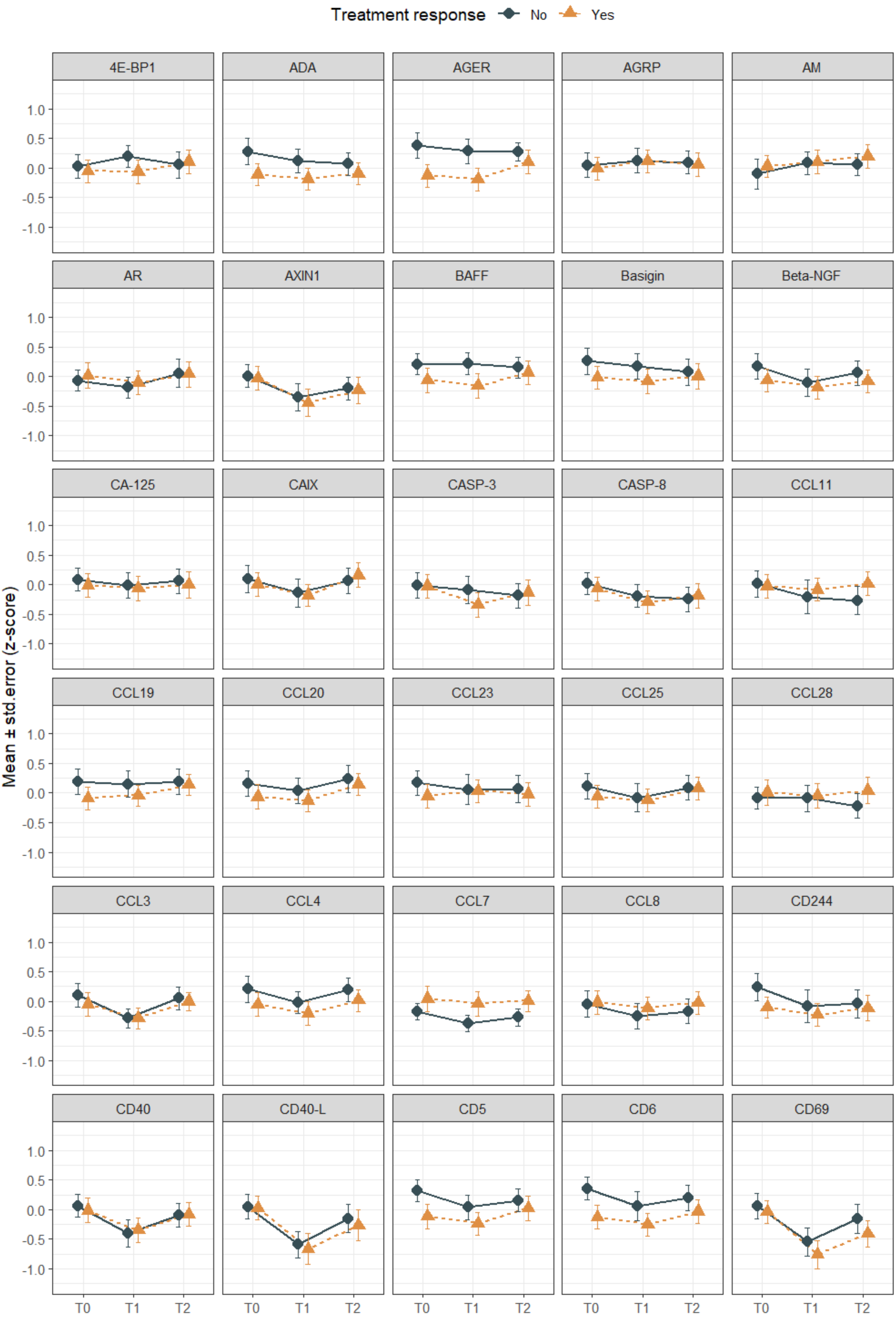

Treatment response
No
Yes
4E-BP1
ADA
AGER
AGRP
AM
AR
AXIN1
BAFF
Basigin
Beta-NGF
CA-125
CAIX
CASP-3
CASP-8
CCL11
CCL19
CCL20
CCL23
CCL25
CCL28
CCL3
CCL4
CCL7
CCL8
CD244
CD40
CD40-L
CD5
CD6
CD69
Mean ± std.error (z-score)
1.0
0.5
0.0
-0.5
-1.0
T0
T1
T2

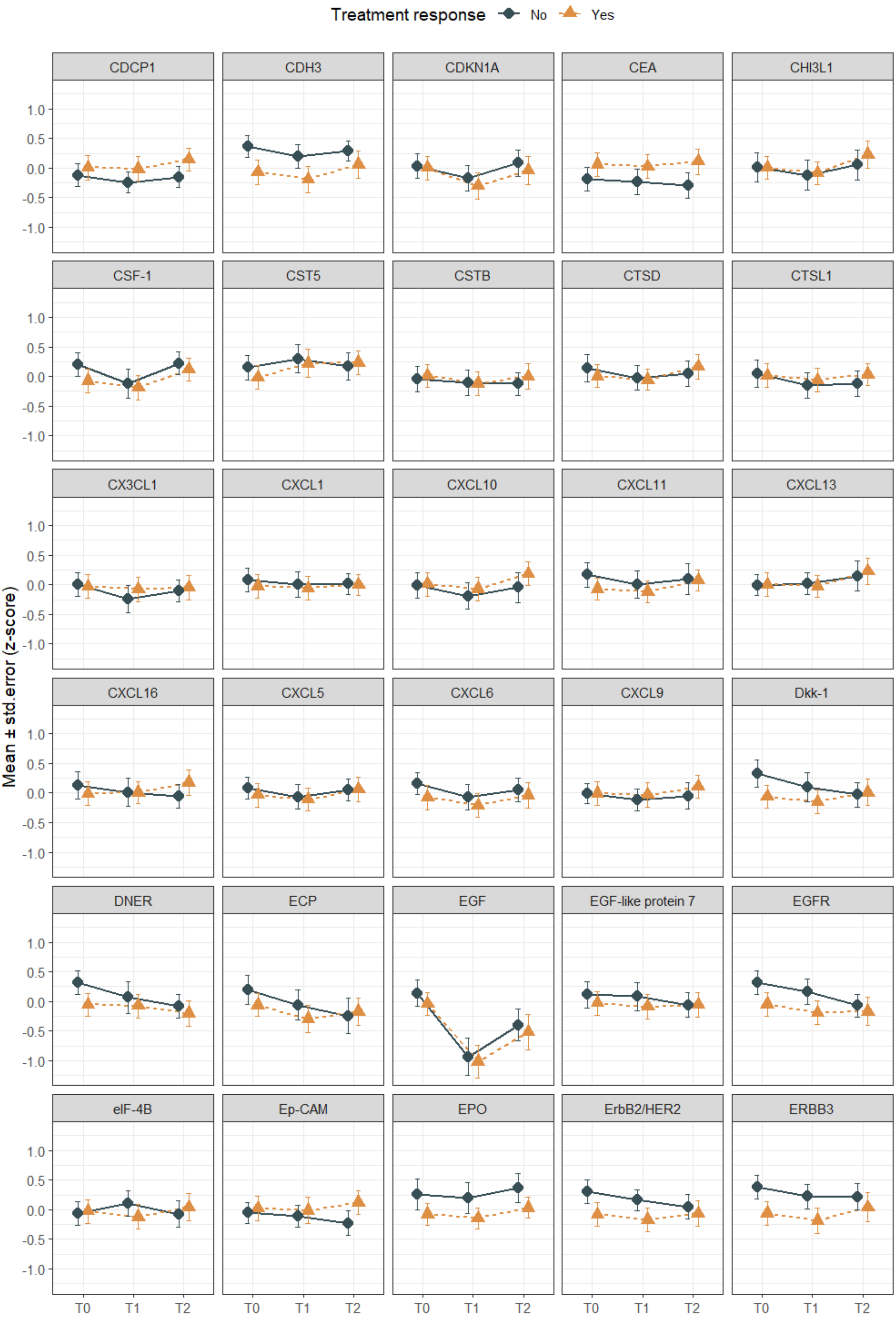

Treatment response
No
Yes
CDCP1
CDH3
CDKN1A
CEA
CHI3L1
CSF-1
CST5
CSTB
CTSD
CTSL1
CX3CL1
CXCL1
CXCL10
CXCL11
CXCL13
CXCL16
CXCL5
CXCL6
CXCL9
Dkk-1
DNER
ECP
EGF
EGF-like protein 7
EGFR
eIF-4B
Ep-CAM
EPO
ErbB2/HER2
ERBB3
Mean ± std.error (z-score)
1.0
0.5
0.0
-0.5
-1.0
T0
T1
T2

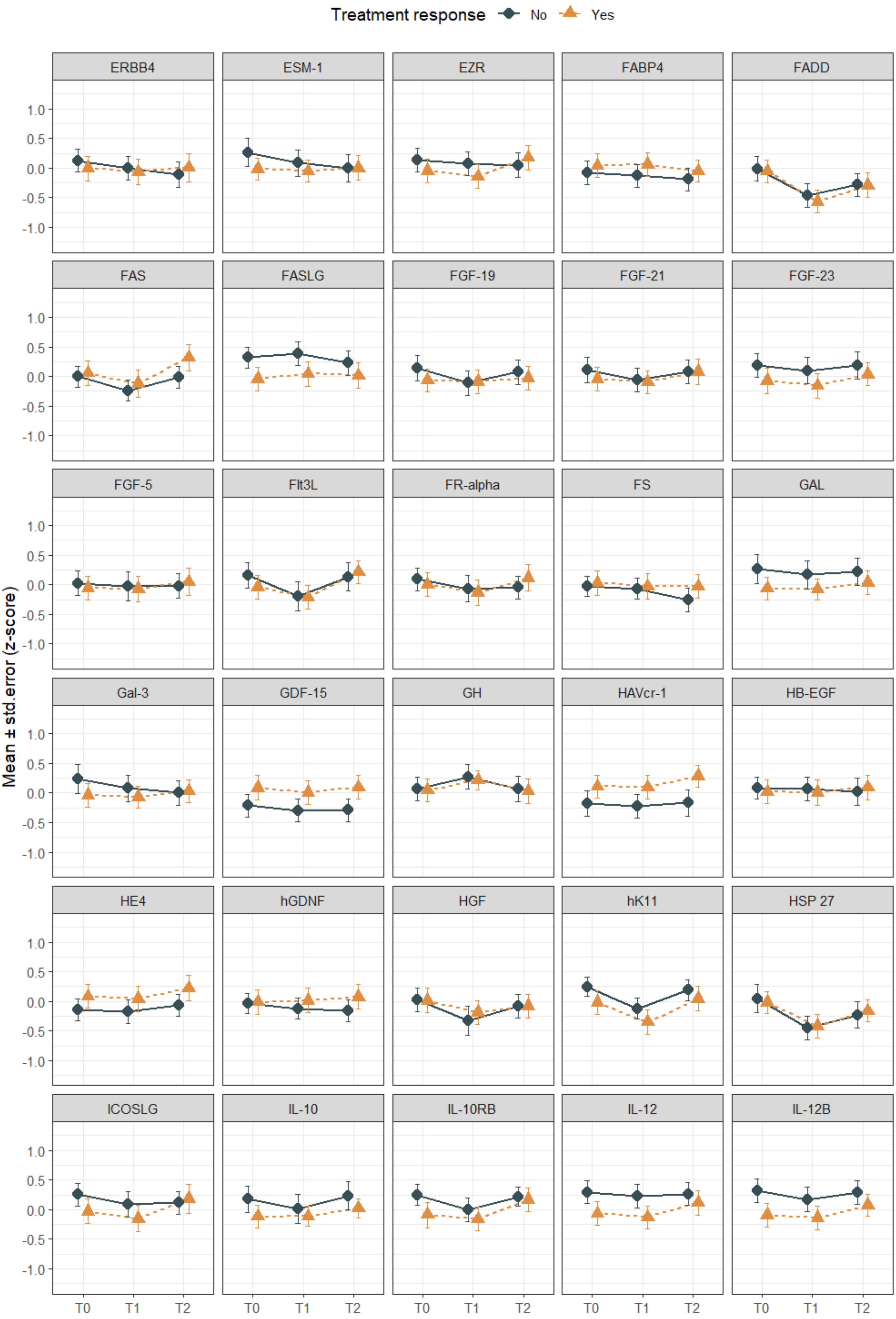

Treatment response
No
Yes
ERBB4
ESM-1
EZR
FABP4
FADD
FAS
FASLG
FGF-19
FGF-21
FGF-23
FGF-5
Flt3L
FR-alpha
FS
GAL
Gal-3
GDF-15
GH
HAVcr-1
HB-EGF
HE4
hGDNF
HGF
hK11
HSP 27
ICOSLG
IL-10
IL-10RB
IL-12
IL-12B
Mean ± std.error (z-score)
T0
T1
T2

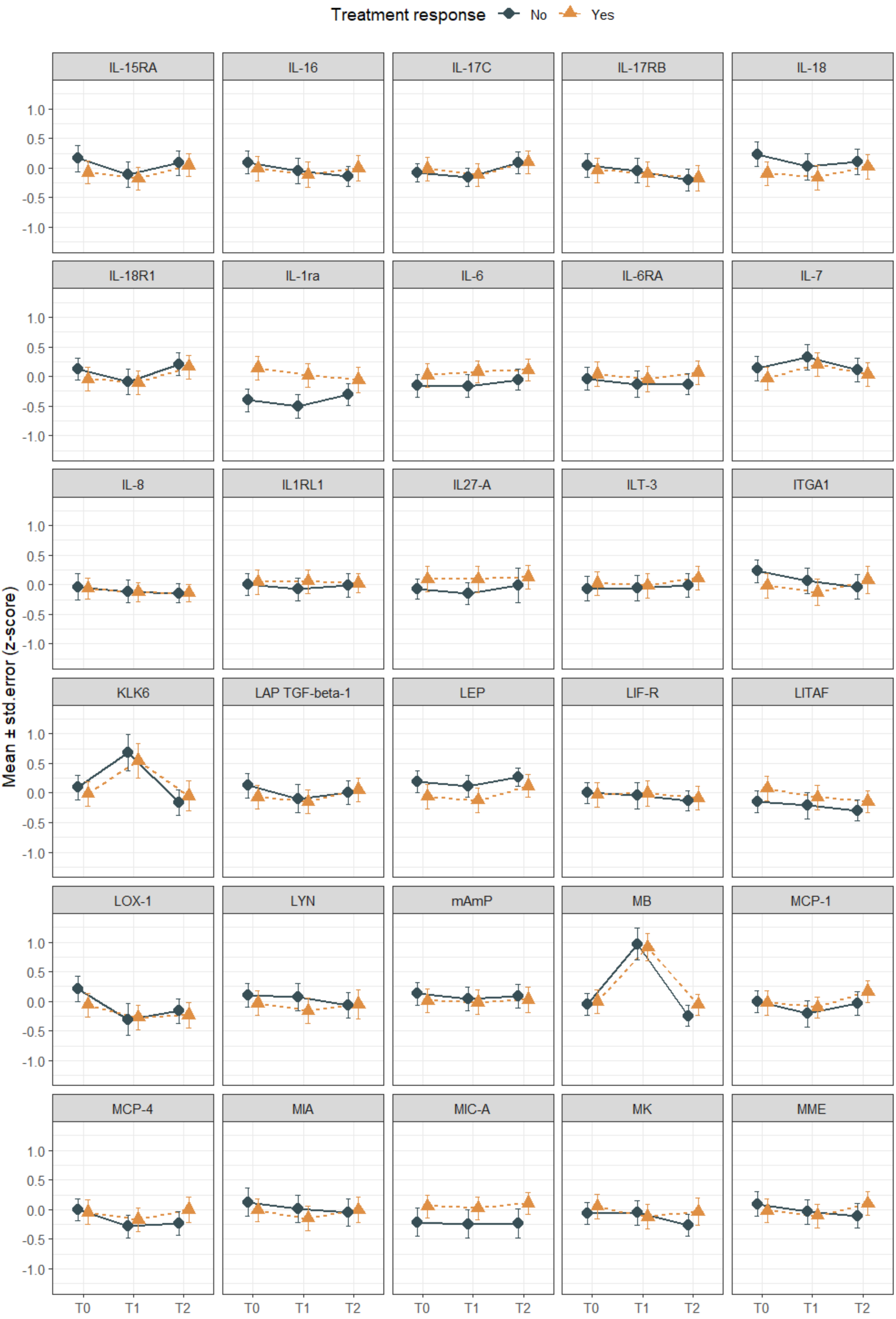

Treatment response
No
Yes
IL-15RA
IL-16
IL-17C
IL-17RB
IL-18
IL-18R1
IL-1ra
IL-6
IL-6RA
IL-7
IL-8
IL1RL1
IL27-A
ILT-3
ITGA1
KLK6
LAP TGF-beta-1
LEP
LIF-R
LITAF
LOX-1
LYN
mAmP
MB
MCP-1
MCP-4
MIA
MIC-A
MK
MME
Mean ± std.error (z-score)
1.0
0.5
0.0
-0.5
-1.0
T0
T1
T2

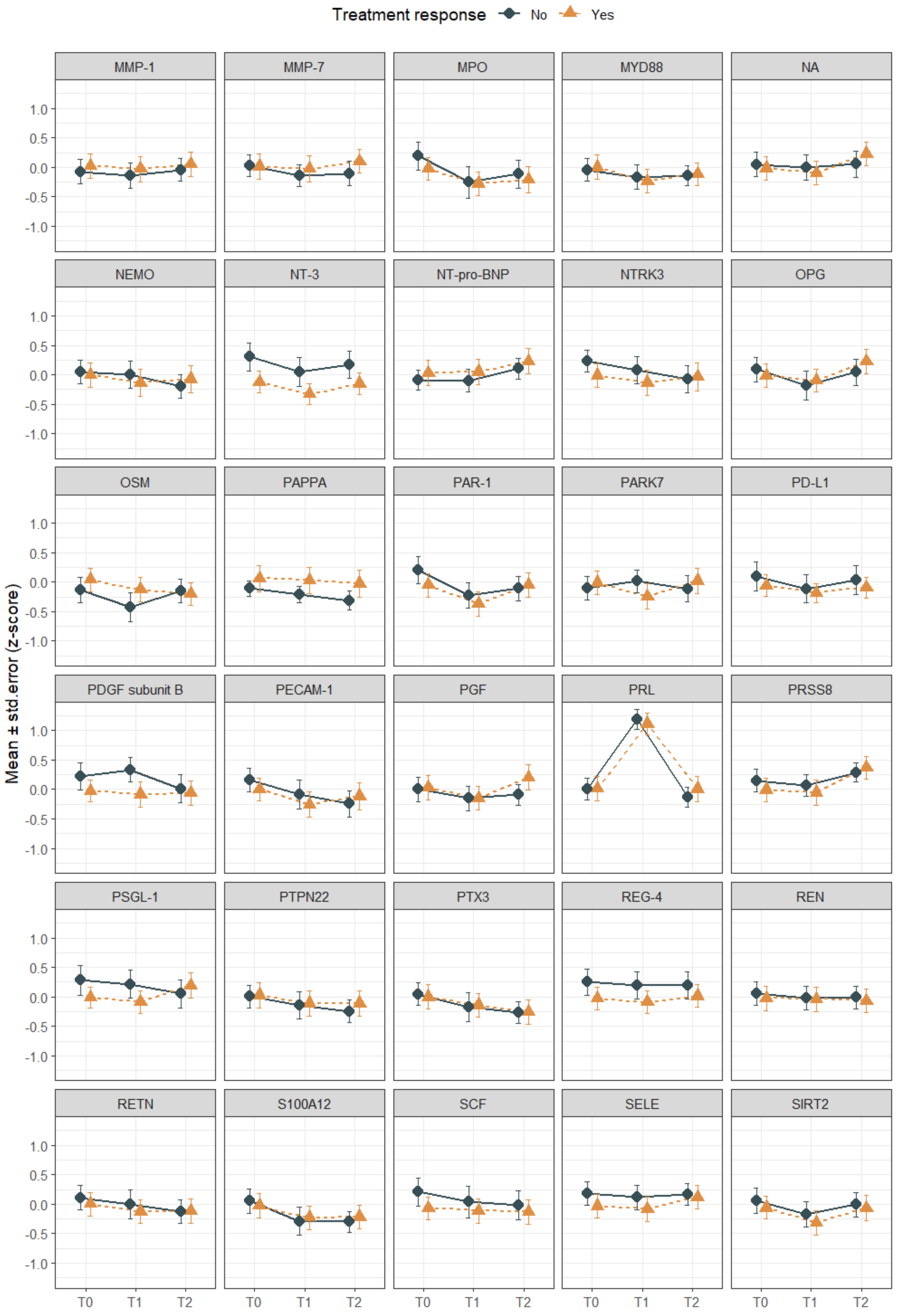
Treatment response
No
Yes
MMP-1
MMP-7
MPO
MYD88
NA
NEMO
NT-3
NT-pro-BNP
NTRK3
OPG
OSM
PAPPA
PAR-1
PARK7
PD-L1
PDGF subunit B
PECAM-1
PGF
PRL
PRSS8
PSGL-1
PTPN22
PTX3
REG-4
REN
RETN
S100A12
SCF
SELE
SIRT2
Mean ± std.error (z-score)
1.0
0.5
0.0
-0.5
-1.0
T0
T1
T2

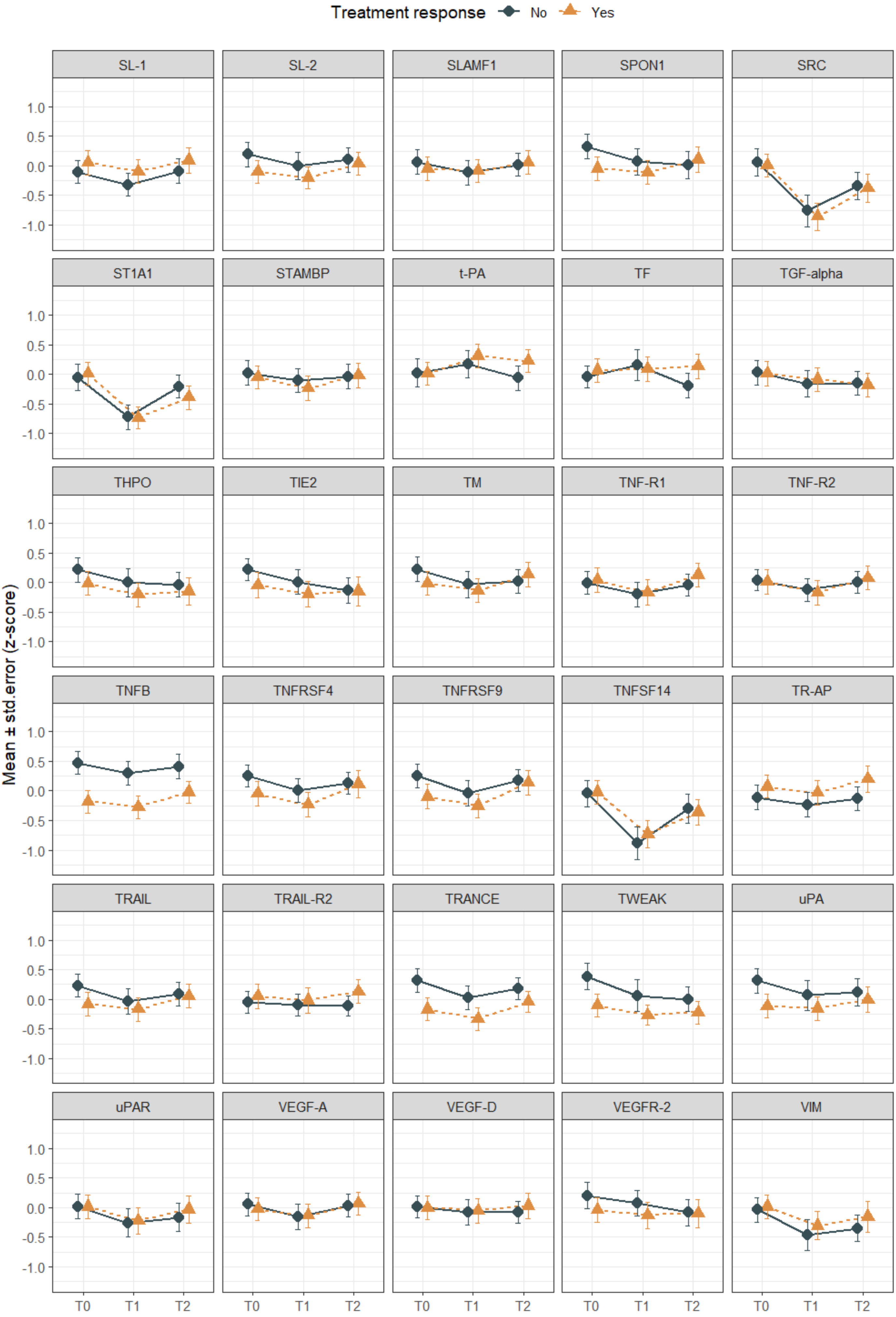

Treatment response
No
Yes
SL-1
SL-2
SLAMF1
SPON1
SRC
ST1A1
STAMBP
t-PA
TF
TGF-alpha
THPO
TIE2
TM
TNF-R1
TNF-R2
TNFB
TNFRSF4
TNFRSF9
TNFSF14
TR-AP
TRAIL
TRAIL-R2
TRANCE
TWEAK
uPA
uPAR
VEGF-A
VEGF-D
VEGFR-2
VIM
Mean ± std.error (z-score)
T0
T1
T2

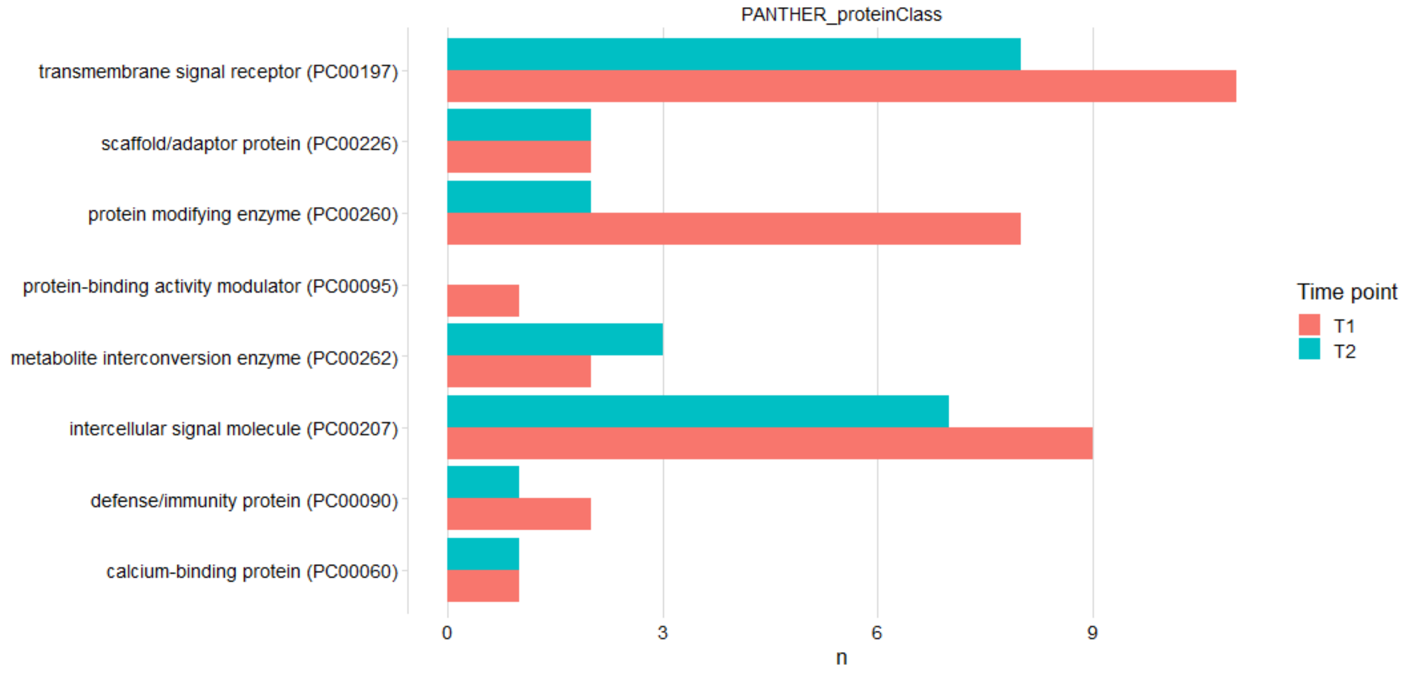
PANTHER_proteinClass
transmembrane signal receptor (PC00197)
scaffold/adaptor protein (PC00226)
protein modifying enzyme (PC00260)
protein-binding activity modulator (PC00095)
metabolite interconversion enzyme (PC00262)
intercellular signal molecule (PC00207)
defense/immunity protein (PC00090)
calcium-binding protein (PC00060)
0
3
6
9
n
Time point
T1
T2

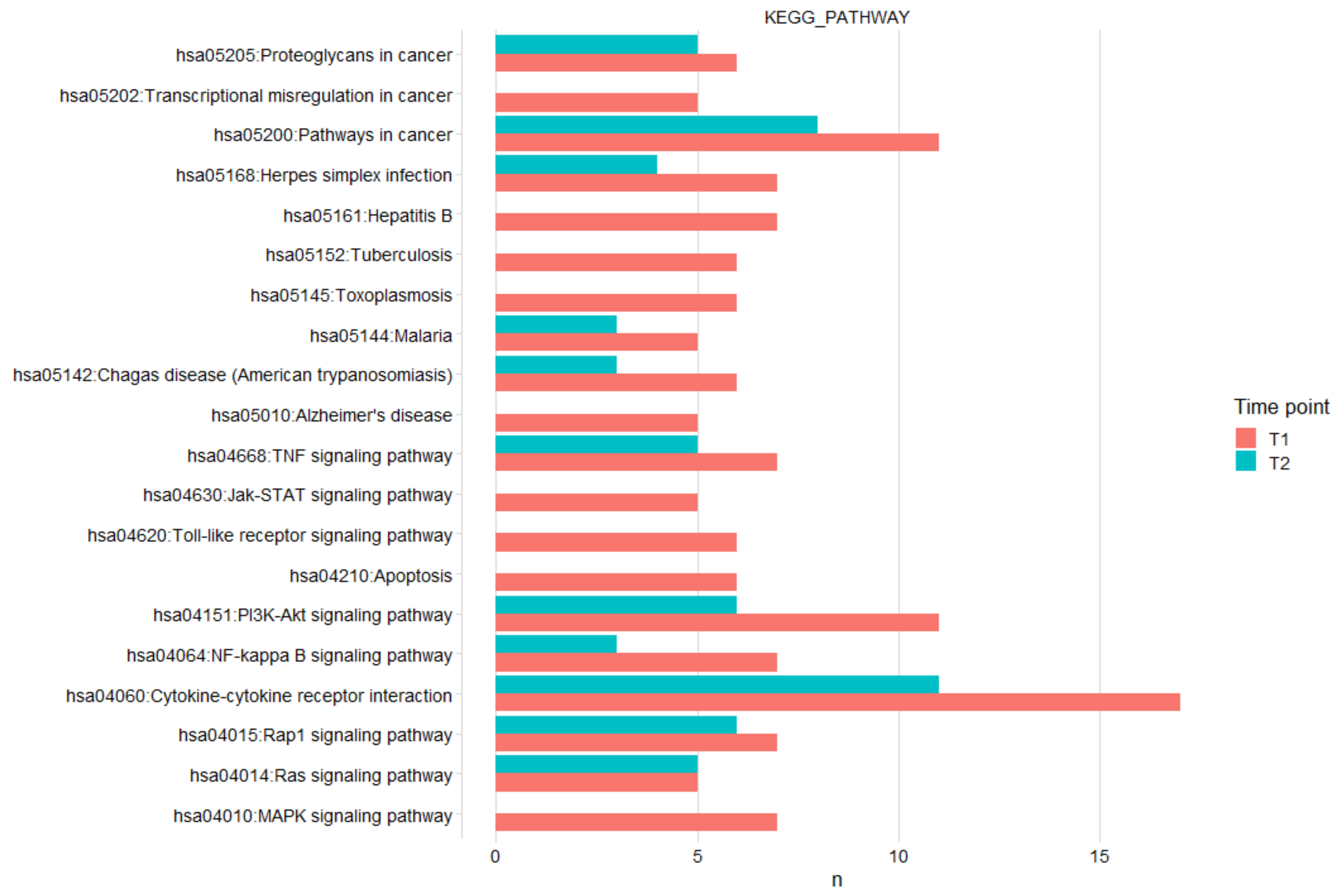
KEGG_PATHWAY
hsa05205:Proteoglycans in cancer
hsa05202:Transcriptional misregulation in cancer
hsa05200:Pathways in cancer
hsa05168:Herpes simplex infection
hsa05161:Hepatitis B
hsa05152:Tuberculosis
hsa05145:Toxoplasmosis
hsa05144:Malaria
hsa05142:Chagas disease (American trypanosomiasis)
hsa05010:Alzheimer's disease
hsa04668:TNF signaling pathway
hsa04630:Jak-STAT signaling pathway
hsa04620:Toll-like receptor signaling pathway
hsa04210:Apoptosis
hsa04151:PI3K-Akt signaling pathway
hsa04064:NF-kappa B signaling pathway
hsa04060:Cytokine-cytokine receptor interaction
hsa04015:Rap1 signaling pathway
hsa04014:Ras signaling pathway
hsa04010:MAPK signaling pathway
0
5
10
15
n
Time point
T1
T2

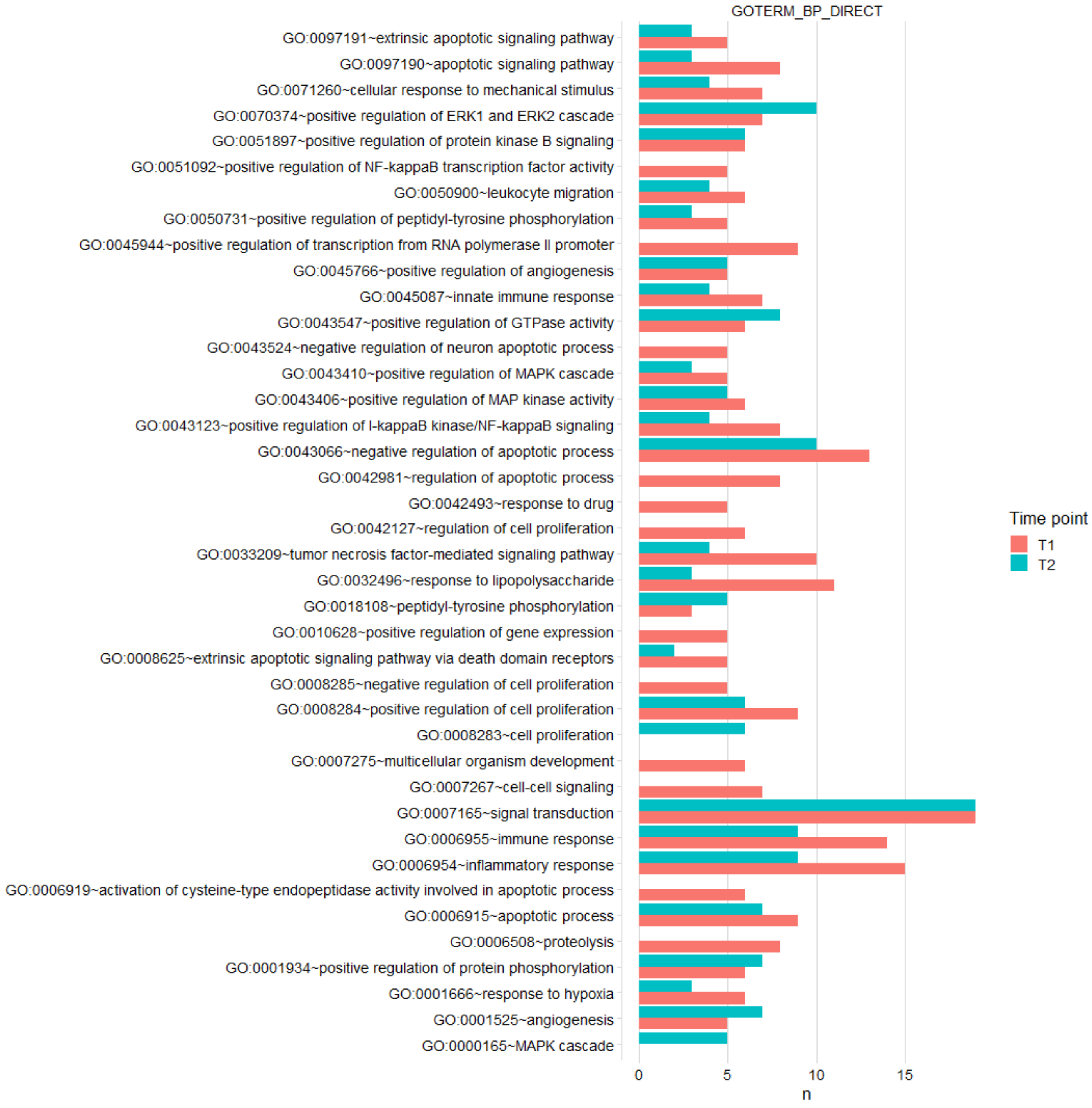
GOTERM_BP_DIRECT
GO:0097191~extrinsic apoptotic signaling pathway
GO:0097190~apoptotic signaling pathway
GO:0071260~cellular response to mechanical stimulus
GO:0070374~positive regulation of ERK1 and ERK2 cascade
GO:0051897~positive regulation of protein kinase B signaling
GO:0051092~positive regulation of NF-kappaB transcription factor activity
GO:0050900~leukocyte migration
GO:0050731~positive regulation of peptidyl-tyrosine phosphorylation
GO:0045944~positive regulation of transcription from RNA polymerase II promoter
GO:0045766~positive regulation of angiogenesis
GO:0045087~innate immune response
GO:0043547~positive regulation of GTPase activity
GO:0043524~negative regulation of neuron apoptotic process
GO:0043410~positive regulation of MAPK cascade
GO:0043406~positive regulation of MAP kinase activity
GO:0043123~positive regulation of I-kappaB kinase/NF-kappaB signaling
GO:0043066~negative regulation of apoptotic process
GO:0042981~regulation of apoptotic process
GO:0042493~response to drug
GO:0042127~regulation of cell proliferation
GO:0033209~tumor necrosis factor-mediated signaling pathway
GO:0032496~response to lipopolysaccharide
GO:0018108~peptidyl-tyrosine phosphorylation
GO:0010628~positive regulation of gene expression
GO:0008625~extrinsic apoptotic signaling pathway via death domain receptors
GO:0008285~negative regulation of cell proliferation
GO:0008284~positive regulation of cell proliferation
GO:0008283~cell proliferation
GO:0007275~multicellular organism development
GO:0007267~cell-cell signaling
GO:0007165~signal transduction
GO:0006955~immune response
GO:0006954~inflammatory response
GO:0006919~activation of cysteine-type endopeptidase activity involved in apoptotic process
GO:0006915~apoptotic process
GO:0006508~proteolysis
GO:0001934~positive regulation of protein phosphorylation
GO:0001666~response to hypoxia
GO:0001525~angiogenesis
GO:0000165~MAPK cascade
0
5
10
15
n
Time point
T1
T2

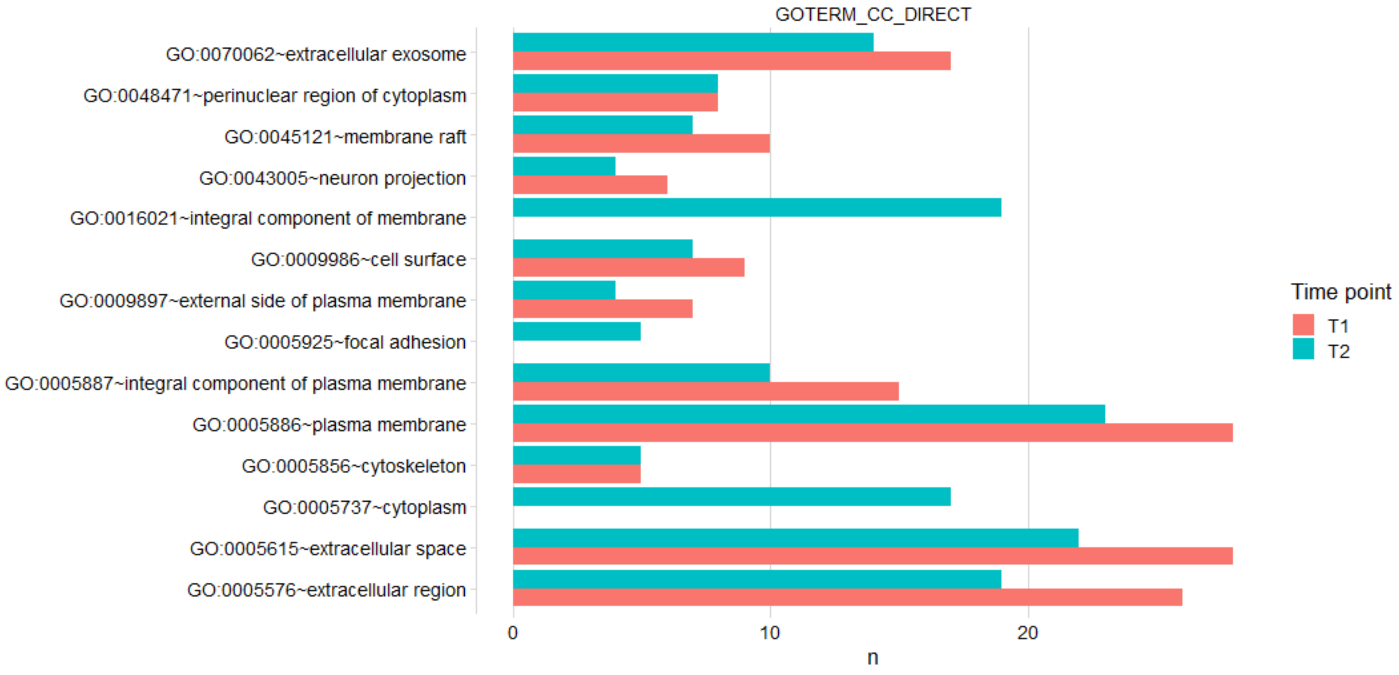
GOTERM_CC_DIRECT
GO:0070062~extracellular exosome
GO:0048471~perinuclear region of cytoplasm
GO:0045121~membrane raft
GO:0043005~neuron projection
GO:0016021~integral component of membrane
GO:0009986~cell surface
GO:0009897~external side of plasma membrane
GO:0005925~focal adhesion
GO:0005887~integral component of plasma membrane
GO:0005886~plasma membrane
GO:0005856~cytoskeleton
GO:0005737~cytoplasm
GO:0005615~extracellular space
GO:0005576~extracellular region
0
10
20
n
Time point
T1
T2

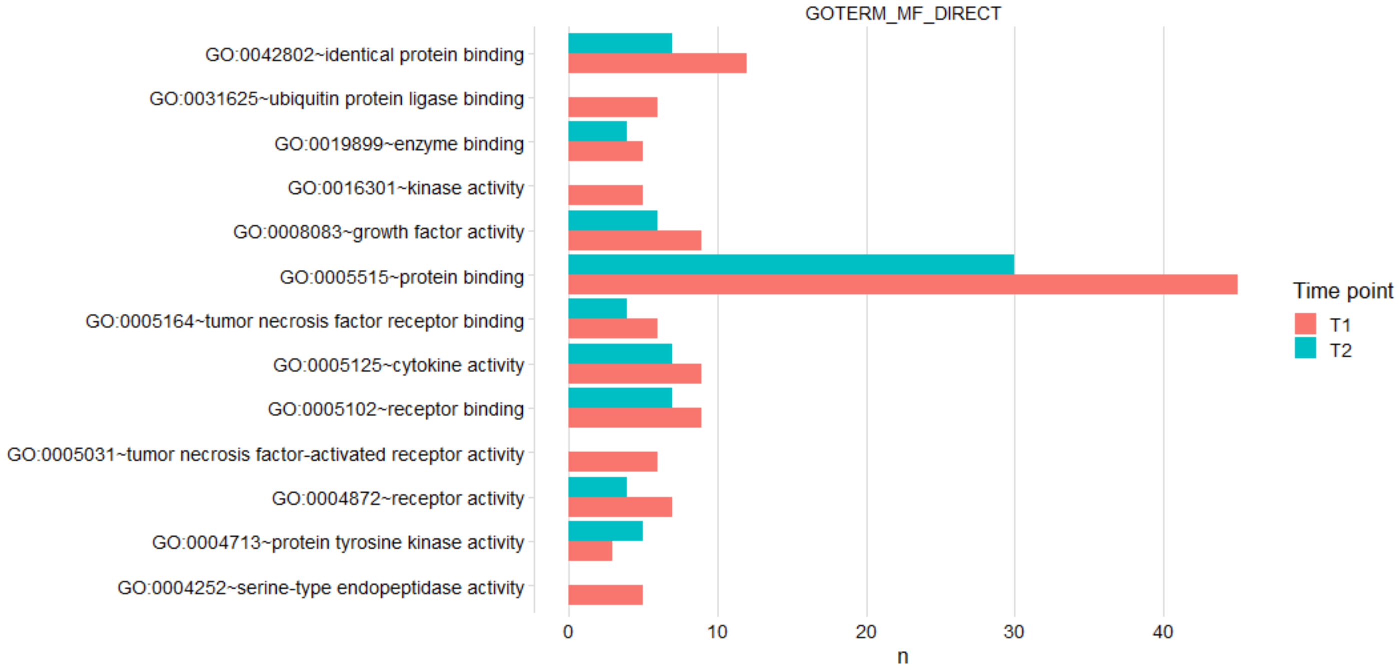
GOTERM_MF_DIRECT
GO:0042802~identical protein binding
GO:0031625~ubiquitin protein ligase binding
GO:0019899~enzyme binding
GO:0016301~kinase activity
GO:0008083~growth factor activity
GO:0005515~protein binding
GO:0005164~tumor necrosis factor receptor binding
GO:0005125~cytokine activity
GO:0005102~receptor binding
GO:0005031~tumor necrosis factor-activated receptor activity
GO:0004872~receptor activity
GO:0004713~protein tyrosine kinase activity
GO:0004252~serine-type endopeptidase activity
0
10
20
30
40
n
Time point
T1
T2
